# Clinical and Surgical Evaluations of Reoperation After Mechanical Mitral Valve Replacement Due to Different Etiologies

**DOI:** 10.3389/fcvm.2021.778750

**Published:** 2022-01-17

**Authors:** Jiehui Li, Shuiyun Wang, Hansong Sun, Jianping Xu, Chao Dong, Min Song, Qibin Yu

**Affiliations:** State Key Laboratory of Cardiovascular Disease, Department of Cardiac Surgery, National Center for Cardiovascular Diseases, Peking Union Medical College, Fuwai Hospital, Chinese Academy of Medical Sciences, Beijing, China

**Keywords:** prosthesis malfunction, mitral valve replacement, perivalvular leakage, thrombus, pannus

## Abstract

**Background:**

This study aimed to evaluate the clinical and surgical characteristics of patients who required reoperation after mechanical mitral valve replacement (MVR).

**Methods:**

We retrospectively identified 204 consecutive patients who underwent reoperation after mechanical MVR between 2009 and 2018. Patients were categorized according the reason for reoperation (perivalvular leakage, thrombus formation, or pannus formation). The patients' medical and surgical records were studied carefully and the rates of in-hospital complications were calculated.

**Results:**

The mean age was 51±12 years and 44% of the patients were male. The reasons for reoperation were perivalvular leakage (117 patients), thrombus formation (35 patients), and pannus formation (52 patients). The most common positions for perivalvular leakage were at the 6–10 o'clock positions (proportions of ≥25% for each hour position). Most patients had an interval of >10 years between the original MVR and reoperation. The most common reoperation procedure was re-do MVR (157 patients), and 155 of these patients underwent concomitant cardiac procedures. There were 10 in-hospital deaths and 32 patients experienced complications. The 10-year survival rate was 82.2 ± 3.9% in general, and the group of lowest rate was patients with PVL (77.5 ± 5.2%). The independent risk factors were “male” (4.62, 95% CI 1.57–13.58, *P* = 0.005) and “Hb <9g/dL before redo MV operation” (3.45, 95% CI 1.13–10.49, *P* = 0.029).

**Conclusion:**

Perivalvular leakage was the most common reason for reoperation after mechanical MVR, with a low survival rate in long term follow-up relatively.

## Introduction

Mechanical prosthesis replacement is a valuable treatment option for mitral valve lesions. It offers a much longer life expectancy than a biological prosthesis. However, a few patients require secondary operation after mechanical mitral valve replacement (MVR). In such cases, the most common pathogeneses include perivalvular leakage (PVL), thrombus, or pannus formation. Although coupled with low prevalence (6.5% for 10 years follow-up, or 1,000–2,000 cases per year in Japan or the US), (1–4) the redo operation remains a significant challenge to clinicians and patients.

Several studies and reviews have examined the therapies and prognosis of redo operations after mechanical MVR, especially for patients with PVL (5–7). However, cases of thrombus or pannus were insufficient. In contrast, it was also worth comprehensively analyzing the operative techniques and their impacts on patients by different etiologies. Therefore, the current study aims to report the clinical features, operative techniques, and complications in-hospital of redo operations after mechanical MVR in our medical center.

## Materials and Methods

### Patients Selection

More than 2,000 mitral valve surgeries have been completed in recent years in our hospital; (8) however, only a few of these patients underwent secondary operations. This retrospective study evaluated patients undergoing reoperation after mechanical MVR from January 2009 to December 2018. Medical records, including echocardiographic, clinical, operative, and in-hospital outcomes, were collected. For inclusion, patients were also required to have undergone transthoracic Doppler echocardiography and preoperative coronary angiography if older than 45. The cause of redo MVR was attributed to perivalvular leakage, thrombogenesis, and pannus formation. Patients were categorized into groups correspondingly. For patients of PVL, because of the variances of mitral annular, a novel item, “summed points of PVL,” was used to evaluate the severity of perivalvular leakage, representing the overall points of a lesion by clock position. The medical records were reviewed carefully to confirm the cause of the operation. The details of lesions were affirmed by the surgery records in cases of inconsistencies with echocardiography or other imaging.

The primary endpoint was defined as the death in the follow-up. The secondary endpoint was defined as the complications in-hospital, including mortality in hospital, bleeding reoperation, continuous renal replacement therapy (CRRT), Intra-Aortic Balloon Pump (IABP), tracheotomy, stroke, ventilator usage ≥96 h, and ICU Stay ≥7 days.

### Statistical Analysis

Continuous variables were expressed as mean ± standard deviation, and categoric variables were represented as percentages. Comparisons were performed by the χ^2^ test or Fisher's exact test for categorical variables and Student's *t*-test for continuous variables. Multiple logistic regression models were used to assess associations with the in-hospital outcomes. As appropriate, survival rates were compared between groups, using the log-rank test or Breslow's test. Predictors or risk factors were analyzed by Cox regression. All variables were included in the univariate analysis. Those with a probability value of <0.1 were included in the multivariate regression. In terms of the Cox multivariate model, missing values were replaced by multiple imputation, and predictor selection using backward stepwise regression with Akaike information criterion (AIC) was performed on all imputed datasets. All statistical tests were two-sided, and a probability value of <0.05 was considered statistically significant. Statistical analysis was performed using IBM SPSS software (version 19.0; IBM Corp., Armonk, NY, USA).

## Results

### Characteristics of Patients With Redo Operations After Mechanical MVR

Two hundred and four patients underwent redo operation after mechanical MVR. Their average height was 1.64 ± 0.1 m, and average weight was 61 ± 11 kg, average age was 51 ± 12 years old, and 50 patients (24.5%) were older than 60. Groups were categorized by etiologies: perivalvular leakage (*n* = 117), thrombus (*n* = 35), and pannus (*n* = 52). The baseline and clinical data of patients enrolled are summarized in Table 1.

**Table 1:** Preoperative characteristics of the study patients.

	PVL^*^(*n* = 117)	**Thrombus (*n* = 35)**	**Pannus (*n* = 52)**	**Overall (*n* = 204)**	***P*-value^†^**
Age (y)	51 ± 13	47 ± 13	52 ± 10	51 ± 12	0.10
Male (%)	69 (59%)	9 (26%)	11 (21%)	89 (44%)	< 0.01
BSA (m^2^)	1.5 ± 0.4	1.2 ± 0.4	1.1 ± 0.3	1.3 ± 0.4	< 0.01
BMI^§^(kg/m^2^)	22 ± 3	23 ± 3	23 ± 4	22 ± 3	0.02
**Risk factors**, ***n*** **(%)**					
Atrial fibrillation^**^	69 (59%)	21 (60%)	36 (69%)	126 (62%)	0.44
Tricuspid regurgitation	74 (63%)	16 (46%)	32 (62%)	122 (60%)	0.17
Pulmonary hypertension	45 (39%)	20 (57%)	25 (48%)	90 (44%)	0.12
Hypertension	17 (15%)	4 (11%)	3 (6%)	24 (12%)	0.26
Diabetes mellitus	6 (5%)	3 (9%)	3 (6%)	12 (6%)	0.75
History of stroke	6 (5%)	1 (3%)	10 (19%)	17 (8%)	< 0.01
NYHA III/IV	69 (59%)	22 (63%)	29 (56%)	120 (59%)	0.80
History of AVR	42 (36%)	9 (28%)	12 (23%)	63 (31%)	0.19
**Echocardiology**					
LVEF, (%)^††^	60 ± 12	52 ± 22	56 ± 15	57 ± 15	0.07
< 50%, n (%)	5 (4%)	1 (3%)	6 (12%)	12 (6%)	0.13
LVEDD	56 ± 13	40 ± 17	45 ± 11	53 ± 10	< 0.01
≥55mm, *n* (%)	74 (63%)	8 (23%)	11 (21%)	93 (46%)	< 0.01
MVPG^§§^	9 ± 5	14 ± 9	11 ± 6	10 ± 6	< 0.01
Hemoglobin (g/dL)	118 ± 23	128 ± 21	120 ± 22	120 ± 23	0.08
Intervals of operations (y)	11 ± 8	9 ± 7	15 ± 8	12 ± 8	< 0.01
≥10y, *n* (%)	61 (52%)	19 (54%)	40 (77%)	120 (59%)	0.01

* *PVL, Perivalvular Leakage*.

† *Comparisons among PVL, thrombus or pannus*.

*BSA, Body Surface Area*.

§ *BMI, Body Mass Index*.

** *Atrial fibrillation, Atrial fibrillation before redo MV operation*.

†† *LVEF, Left Ventricular Ejection Fraction*.

*LVEDD, Left Ventricular End Diastolic Diameter*.

§§ *MVPG, Mitral Valve Pressure Gradient*.

*P value < 0.05, with significant difference in statistics*.

Most baseline characteristics among the patients showed no difference in statistical terms. Patients with perivalvular leakage had more proportion of males (*n* = 69, 59%), with the highest BSA (1.5 ± 0.4 m^2^) and the lowest BMI (22 ± 3 kg/m^2^). The pannus group had the longest interval of operations (15 ± 8 years, *p* = 0.001). Group PVL had the largest left ventricular end-diastolic diameter (LVEDD, 56 ± 13 mm, *p* < 0.001), and group thrombus had the greatest mitral valve pressure gradient (MVPG, 14 ± 9 mmHg, *p* = 0.004).

**Figure 1:**
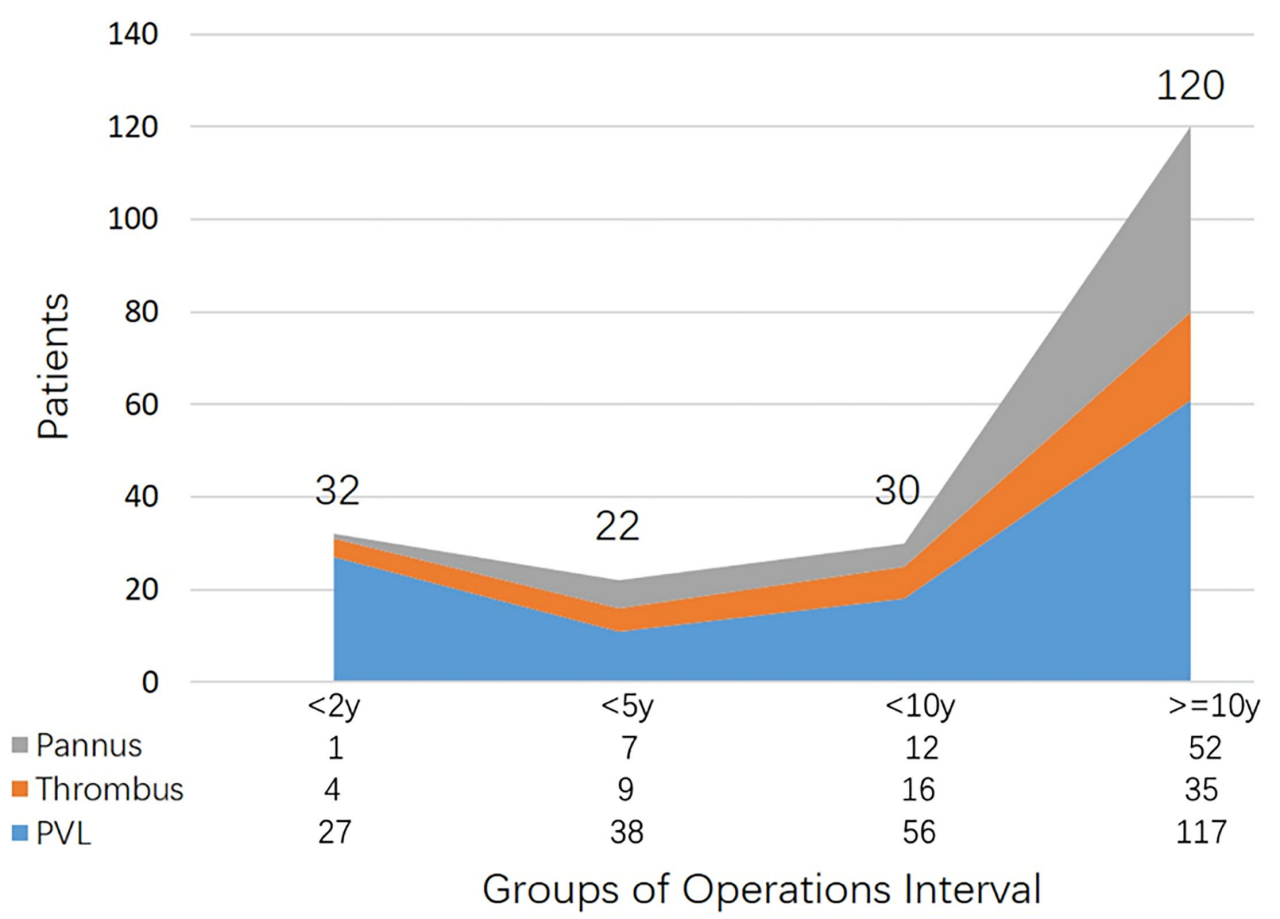
Patient distributions of etiologies categorized by operations interval.

As displayed in Figure 1, more than half of the patients had more than 10 years gap between operations. Especially for patients with pannus, the average interval was 15 ± 8 years, and few of them underwent redo operations <10 years after primary surgery. The majority of PVL patients were in the group “ < 2 years” (*n* = 27, 84.4%). Moreover, among patients with thrombus, the morbidity of redo surgery appeared to be similar in the entire cohort.

**Figure 2:**
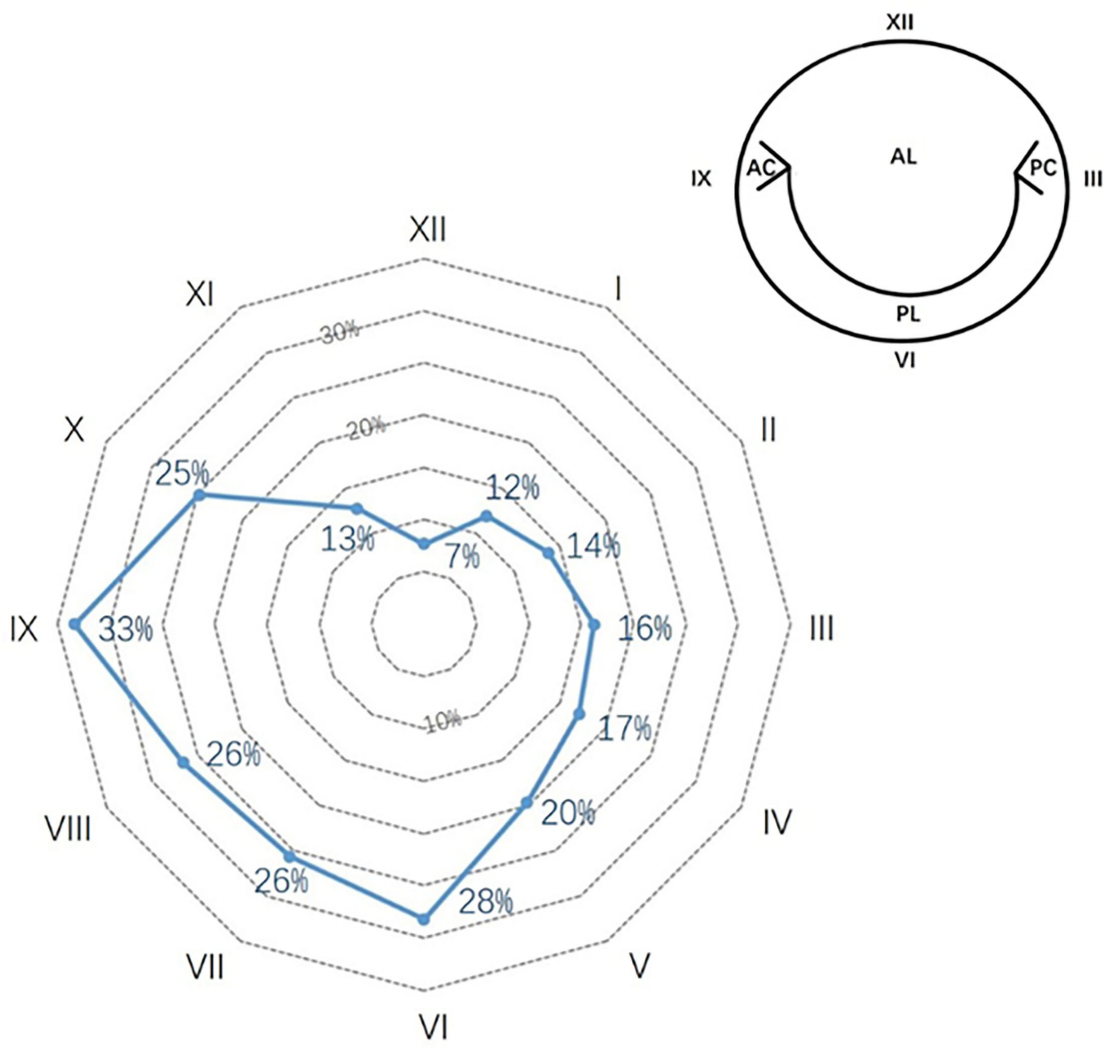
Perivalvular leakage of mechanical mitral prosthesis in the native valve anatomy. The percentages show the prevalence in each clock position. The most frequent positions were from 6 to 10 o'clock.

Detailed documentation of perivalvular leakage was available for 109 (93% of PVL group). Figure 2 illustrates the distribution of perivalvular leakage illustrated by the clock position on a radar chart. Eighty-one patients (74% of PVL records) had more than one point of lesion. The most susceptible regions appeared from six to ten o'clock, with a prevalence of no <25%.

**Figure 3:**
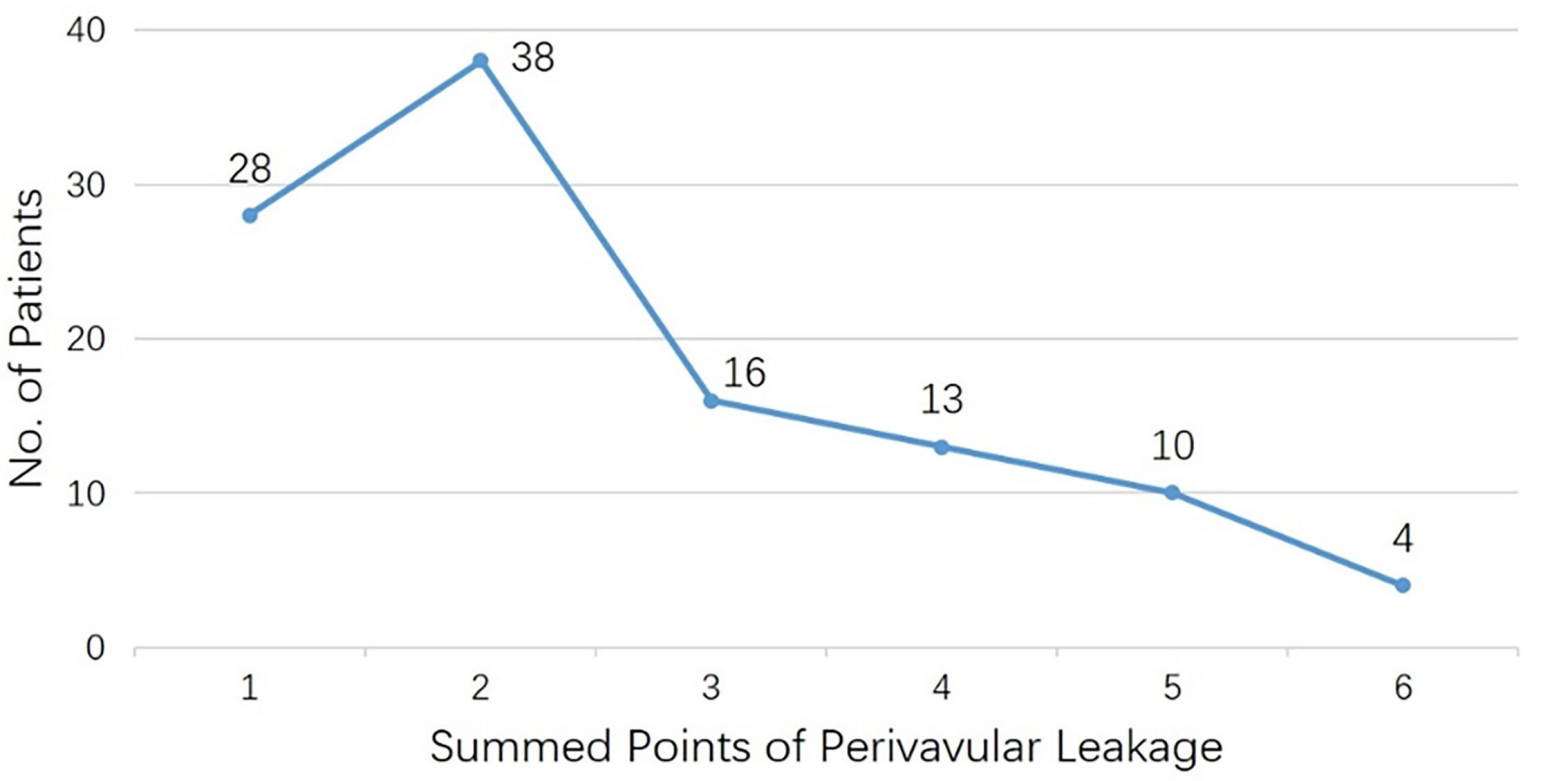
Patients number for each subgroup with summed points of perivalvular leakage.

We used a novel item, “summed points of PVL,” to evaluate the severity of perivalvular leakage, representing the lesion's overall points by clock position. Generally, patients were found to have 2.6 ± 1.4 points (Figure 3), and it had no relationship with the interval of operations (r = 0.06, *p* = 0.56). Furthermore, we analyzed the PVL patients combined with AVR simultaneously and found no relationship between “summed points of PVL” and “aortic valve replacement” (eight of one point, eight of two summed points, and seven of more than two points, *p* = 0.797).

### Characteristics of Redo Operations After Mechanical MVR

One hundred and fifty-seven patients underwent repeat MVR, with 147 mechanical prostheses (94% of re-MVR) implanted. Forty patients of perivalvular leakage (34% of PVL group) underwent PVL repair, and seven patients (14% of pannus group) underwent pannus clearance only.

One hundred and fifty-five patients underwent concomitant heart procedures, including 131 tricuspid valve repairs and 54 aortic valve replacements (AVR) (Table 2). The operation time was 327 ± 104 min, and the aortic cross-clamping (ACC) time was 103 ± 48 min. Operations in the PVL group had a shorter ACC time than non-PVL groups (min: 96 ± 46: 112 ± 50, *p* = 0.01). We had recorded 10 deaths (mortality of 4.9%) in the hospital. And as shown in Table 3, there was no difference among the three groups in terms of complications in general or in detail.

**Table 2:** Blood transfusion and operative characteristics of the study patients.

	PVL^***^(*n* = 117)	**Thrombus (*n* = 35)**	**Pannus (*n* = 52)**	**Overall (*n* = 204)**	***P*-value^†††^**
**Operative techniques**					
PVL-repairing	40 (34%)	-	-	40 (20%)	-
Pannus clearance	-	-	7 (14%)	7 (3%)	-
Mitral valve replacement	77 (66%)	35 (100%)	45 (87%)	157 (77%)	<0.01
Mechanical prosthesis^§§§^	76	30	41	147	0.02
Size of prosthesis (mm)	27 ± 2	26 ± 1	26 ± 1	26 ± 2	<0.01
Concomitant procedure, n^****^	87 (74%)	24 (69%)	44 (85%)	155 (76%)	0.19
Aortic valve replacement	26 (22%)	7 (20%)	21 (40%)	54 (27%)	0.03
Tricuspid valve repairing	76 (65%)	20 (57%)	35 (67%)	131 (64%)	0.61
Operation time (mins)	330 ± 107	321 ± 114	325 ± 98	327 ± 104	0.93
CPB time^††††^	149 ± 72	157 ± 71	161 ± 65	153 ± 70	0.56
ACC time	96 ± 46	105 ± 45	117 ± 53	103 ± 48	0.02
P-ACC time^§§§§^	30 ± 22	33 ± 30	40 ± 52	33 ± 34	0.89
P-ACC: ACC	0.36 ± 0.27	0.34 ± 0.29	0.36 ± 0.38	0.35 ± 0.30	0.33
Length of hospital stay (d)	26 ± 18	15 ± 7	18 ± 10	22 ± 15	<0.01
Postoperative stay (d)	13 ± 11	11 ± 6	10 ± 6	12 ± 9	0.08
ICU stay (h)	101 ± 147	94 ± 116	83 ± 99	95 ± 131	0.76
Ventilator usage (h)	48 ± 87	46 ± 76	37 ± 39	45 ± 75	0.38
**Blood Transfusion**					
Red blood cell (u)	8 ± 7	9 ± 14	6 ± 5	8 ± 8	0.49
Plasma (ml)	917 ± 780	1,216 ± 2,229	741 ± 572	939 ± 1163	0.63
Platelet (u)	1.6 ± 1.5	1.5 ± 0.8	1.5 ± 1.6	2.0 ± 3.5	0.89

*** *PVL, Perivalvular Leakage*.

††† *Comparisons among PVL, thrombus or pannus*.

*Values are n (%), or median (interquartile range)*.

§§§ *Comparisons (PVL, Thrombus, & Pannus) in patients received redo MV replacement*.

**** *No. of patients received concomitant procedures*.

†††† *CPB, Cardio-Pulmonary Bypass*.

*ACC, Aortic Cross Clamping*.

§§§§ *P-ACC, CPB time of Post ACC*.

*P value < 0.05, with significant difference in statistics*.

**Table 3:** Complications of redo operations after mechanical MVR^*^.

	**PVL^†^(*n* = 117)**	**Thrombus (*n* = 35)**	**Pannus (*n* = 52)**	**Overall (*n* = 204)**	***P-*value**
Complications, n (%)^§^	19 (16%)	6 (17%)	7 (13%)	32 (16%)	0.87
ICU stay ≥ 7d	12 (10%)	3 (9%)	2 (4%)	17 (8%)	0.38
Ventilator usage ≥ 96 h	7 (6%)	3 (9%)	4 (8%)	14 (7%)	0.84
Mortality in hospital	4 (3%)	3 (9%)	3 (6%)	10 (5%)	0.44
CRRT^**^	8 (7%)	3 (9%)	1 (2%)	12 (6%)	0.35
Tracheotomy	5 (4%)	1 (3%)	1 (2%)	7 (3%)	0.73
Bleeding reoperation	5 (4%)	2 (6%)	4 (8%)	11 (5%)	0.66

* *Complications: mortality in hospital, bleeding reoperation, CRRT, IABP, tracheotomy, stroke, ventilator usage ≥ 96 h, ICU Stay ≥ 7 d;*

† *PVL, Perivalvular Leakage*.

*Comparisons among PVL, thrombus or pannus*.

§ *No. of patients with complications after redo MV operation*.

** *CRRT, Continuous Renal Replacement Therapy*.

### Follow-Up of Redo Operations After Mechanical MVR

**Figure 4:**
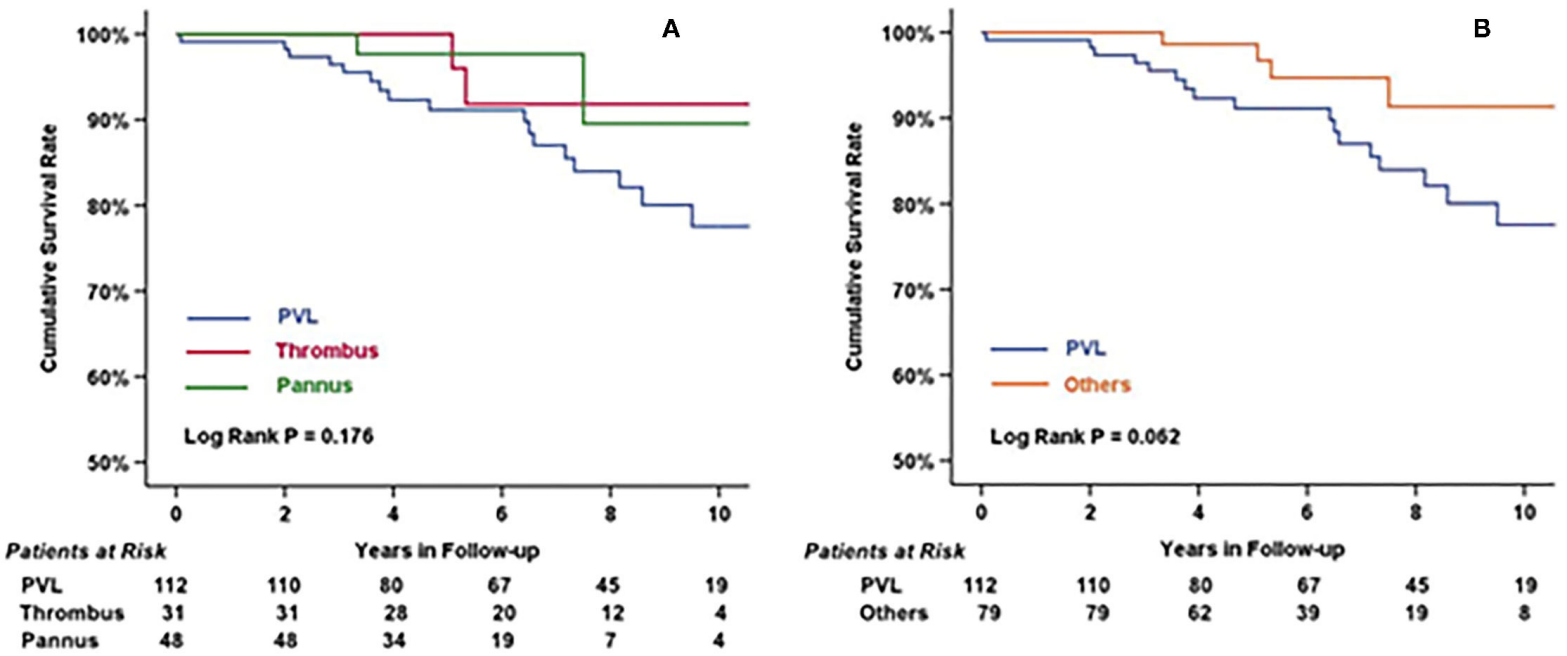
Cumulative survival rate of patients categorized by etiologies **(A)** and by PVL/Others **(B)**. There was no statistical difference among three groups (log rank *P* = 0.176), but had significant difference between patients with or without perivalvular leakage before redo MV operation (log rank = 0.062).

The overall survival rate at 10 years was 82.2 ± 3.9%. As shown in Figure 4, there was no difference among the three groups in terms of etiologies (log-rank test, *P* = 0.18). However, differences could be observed after categorizing patients into the PVL group and Others group (non-PVL group) (log-rank test, *P* = 0.06). Univariate and multivariate Cox regression analyses (Table 4) revealed that “Male” (4.62, 95% CI 1.57–13.58, *P* = 0.005) and “Hb <9g/dL before secondary MV operation” (3.45, 95% CI 1.13–10.49, *P* = 0.029) were the independent risk factors in patients undergoing redo operations after mechanical MVR. Additionally, LVEF might influence the late outcomes of patients after the redo MV operation (1.07, 95% CI 1–1.14, *P* = 0.061).

**Table 4:** Univariate/multivariate cox regression of redo operations after mechanical MVR.

**Variables**	**Univariate regression HR (95% CI)**	***P-*value**	**Multivariate regression HR (95% CI)**	***P-*value**
Age	1.02 (0.98, 1.06)	0.256		
Male	4.62 (1.69, 12.63)	0.003	4.62 (1.57, 13.58)	0.005
BMI^xxii^	1.02 (0.90, 1.16)	0.728		
Etiologies^xxiii^	0.54 (0.26, 1.11)	0.093	0.84 (0.41, 1.76)	0.649
NYHA III/IV	1.41 (0.57, 3.50)	0.457		
PH^xxiv^	0.91 (0.38, 2.20)	0.839		
Atrial fibrillation^xxv^	1.19 (0.48, 2.94)	0.712		
Hb <9g/dL	3.67 (1.22, 11.06)	0.021	3.45 (1.13, 10.49)	0.029
MVR^xxvi^	0.60 (0.24, 1.49)	0.269		
Multi-Operation^xxvii^	3.12 (0.73, 13.38)	0.126		
LVEDD^xxviii^	1.01 (0.97, 1.05)	0.770		
LVEF^xxix^	1.06 (1, 1.13)	0.063	1.07 (1, 1.14)	0.061

xxii *BMI, Body Mass Index*.

**xxiiii:** *Etiologies, PVL, thrombus, pannus*.

xxiv *PH, Pulmonary Hypertension*.

xxv *Atrial fibrillation, Atrial fibrillation before redo MV operation*.

xxvi *MVR, Patients receiving MVR finally during the redo MV operation*.

**xxvii:** *Multi-Operation, Multi-cardiac operations performed simultaneously during the redo MV operation*.

**xxviii:** *LVEDD, Left Ventricular End Diastolic Diameter*.

xxix *LVEF, Left Ventricular Ejection Fraction*.

## Discussion

### Patients by Different Etiologies of Redo Operations After Mechanical MVR

In the present study, perivalvular leakage was the most important pathogenesis leading to a secondary operation after mechanical MVR. Patients with perivalvular leakage had a longer diameter of left ventricular end-diastole, and the percentage of left ventricular dilation (LVEDD ≥55 mm) was up to 63%. This could be attributed to the fact that the pathophysiology was similar to mitral regurgitation, which also led to a shorter interval between operations than patients with pannus or thrombus (years: 11 ± 8: 13 ± 8, *p* = 0.058). These findings were in line with Botta et al. report (mean: 130 months) (9). Most studies proposed that patients require intervention only with the moderate-severe PVL or coupled with symptoms; mild PVL without symptoms usually had a benign course and could be managed in follow-up carefully (10). However, the surgeries peaked slightly in PVL patients during the first 2 years. This spike in the extremely short interval might be attributed to the surgical techniques being used. Meanwhile, patient numbers in other groups increased gradually with time in 10 years, after former mechanical MVR from our study.

In the current investigation, the perivalvular leakage in the mitral position was predominantly at six to ten o'clock position (≥25% in each point), which was mirror-symmetric to Bouhout et al. study (from two to six o'clock, ≥35% for each point) (6). Meanwhile, another important assessment was the severity of PVL. Because of the different sizes of mitral prostheses, it might be unreasonable to compare them by length or width directly. We analyzed the item, “summed points of PVL” (2.6 ± 1.4 points), which represented the overall leakage points by clock position. These data could benefit further analysis.

Patients with pannus had the greatest MVPG in the cohort (14 ± 9 mmHg, *p* = 0.004). This was in line with previous studies and reports on pannus and thrombus (11, 12). The current consensus showed that pannus always formed as circular mass curved along the ring, and thrombus tended to infest into the prosthesis, which further restricted leaflet motion and caused a malfunction. Moreover, patients with pannus had the most prolonged interval of operations (15 ± 8 years, *p* = 0.001). The development of pannus that can affect the hemodynamics of artificial prostheses requires a significant amount of time (13). In contrast, thrombi always give rise to acute symptoms, leading to shorter intervals (9 ± 7 years), similar to our study. These findings were consistent with Separham et al. report (14).

### Techniques and Details of Redo Operations

Thirty-three percent of PVL patients underwent perivalvular leakage repair, with shorter aortic cross-clamping time than redo MVR in the same group (min: 78 ± 45: 105 ± 44, *p* = 0.002). However, this procedure did not cut down the operation time and Cardio-Pulmonary Bypass (CPB) time in general. Moreover, CPB time after ACC was not significantly different between the three etiology groups. Thus, it could be recognized that the operative techniques would not impact the difficulty of surgery.

The most common type of operative technique was the repeated replacement of mitral prosthesis (re-MVR), received by 66% of patients of PVL in our cohort. However, Bouhout et al. study showed that 76% of patients underwent PVL repair, who also reported the opposite distribution of PVL points mentioned above (6). PVL patients also had a larger prosthesis size than patients with thrombus or pannus in our hospital. All re-MVRs implanted artifacts had a size similar to the previous operation. The mechanical prosthesis was the mainstream choice for surgeons, even for patients aged more than 65 years. There were 11 cases of 14 implantations (three bioprosthetic valves). For these cases, there were four with small aortic annulus (size 19 or 21), two with endocarditis, one with Behcet's disease, and one with patient preference. These results might differ from Fukunaga et al. findings, which suggested that 75% of patients aged 50–69 years had received biological prostheses (15). However, the composition of their cohort was significantly different from ours, as there were only 6.8% cases of PVL in the mechanical MVR group.

Among patients undergoing concomitant operations, 22% (54 patients) had AVR, of which 31 patients underwent secondary replacement. The most common lesion of the aortic prosthesis was pannus formation (*n* = 16), and there were eight patients with a mean aortic valve pressure gradient >30 mmHg at rest. In our cohort, secondary AVR was more common among patients with a history of AVR [OR 3.54, 95%CI (1.84, 6.81), *p* < 0.01].

Meanwhile, in the present study, there were seven patients who underwent pannus removal through the transaortic route. Their MVPG was 5 ± 1 mmHg, compared to 11 ± 7 mmHg (*p* = 0.001). Park et al. (16) found that patients with MVPG <5 mmHg could not benefit from the pannus clearance. Our findings were in line with their report.

### Endpoints After Redo Operations

Complications during the perioperative period have already been outlined in the Results section. The in-hospital mortality in our cohort was relatively low in our study compared to previous reports (2, 7, 17). The prevalence of dialysis and postoperative stroke (one case) was at an extremely low level, (18, 19) and the morbidity of bleeding during reoperation in our study was similar to a previous study (20). However, the ICU stay time and length of hospital stay after the operation was longer in our study than that reported earlier (19).

The overall survival rate at 10 years for our study participants was 82.2 ± 3.9%, similar to that reported by Fukunaga et al. (survival rate: 79.2%) (21). In our study cohorts, the PVL group had an outcome of 77.5 ± 5.2% after a 10-year follow-up which was the worst among the three groups. This might be due to the larger LVEDD than other groups (percentage of LVEDD>65 mm; PVL: Thrombus: Pannus; 63: 23: 21%). Patients with PVL always had worse outcomes than those patients with thrombus or pannus (1, 7, 14, 21). However, the survival rate of PVL patients in our study was still higher than in some studies, such as Bouhout et al. (57 ± 6%) (6). This may be attributed to the inclusion of relatively older adults in their group and a higher proportion of patients with NYHA III/IV. Furthermore, Botta et al. reported a survival rate at 5 years similar to our study (88.8: 84.0%) (9). However, these studies had different outcomes after Cox regression analysis. Our research had found that “male” and “Hb <9g/dL before secondary MV operation” were independent risk factors affecting long-term prognosis.

Patients with thrombus in our study had a 10 years survival rate of 91.8 ± 5.5%, which was mildly lower than that reported by Raman et al. (97.4 ± 1.2%) (22). Furthermore, those with pannus had a moderate survival rate (89.5%) compared with the thrombus group and PVL group, similar to findings reported by Park et al. (87.6%) (23). This suggests that patients with mechanical mitral valve dysfunction would have a good prognosis if they underwent a proper redo procedure.

### Limitations of the Study

There were some limitations to our study. First, due to insufficient or missing clinical data, the insights drawn based on the surgical and patient history might not be fully valid and acceptable. Many patients did not undergo echocardiography in recent 2–3 years, making it further challenging to analyze the functioning of mechanical prostheses.

## Conclusion

Despite the low prevalence, reoperation after mechanical mitral valve replacement remains a key challenge. In our study, perivalvular leakage might be the possible cause of operation, which might lead to a low survival rate in the long-term follow-up relatively. Male patients with low hemoglobin (Hb <9g/dL) before the redo MV operation might have a worse outcome during the long-term follow-up compared to other patient subgroups.

## Data Availability Statement

The original contributions presented in the study are included in the article/supplementary material, further inquiries can be directed to the corresponding author.

## Ethics Statement

The studies involving human participants were reviewed and approved by the Ethics Committee of Fuwai Hospital, National Center for Cardiovascular Disease. The Ethics Committee waived the requirement of written informed consent for participation.

## Author Contributions

SW, HS, JX, and CD: main surgeons of operations. JL, MS, and QY: statistics. JL and SW: writing. SW: general responsibility. All authors contributed to the article and approved the submitted version.

## Conflict of Interest

The authors declare that the research was conducted in the absence of any commercial or financial relationships that could be construed as a potential conflict of interest.

## Publisher's Note

All claims expressed in this article are solely those of the authors and do not necessarily represent those of their affiliated organizations, or those of the publisher, the editors and the reviewers. Any product that may be evaluated in this article, or claim that may be made by its manufacturer, is not guaranteed or endorsed by the publisher.
